# Alphabet of Scientific Chemistry, for the Use of Beginners

**Published:** 1834-01-01

**Authors:** 


*Alphabet of Scientific Chemistry, for the Use of Beginners.* By JAMES RENNIE, M.A., Professor of Zoology, King's College, London, &c.—London, 1833. 8vo.

THE merest novice in chemistry cannot but perceive, on examination, that the errors contained in this little work are almost as numerous as the lines in which it is comprised: had it therefore been written by any one but a professor of a college, we should have thrown it amongst our waste paper almost at the first glance, as a thing beneath criticism.

The volume before us opens with what is called *a plan of the work,* in which, after an attentive perusal, we find no plan given. This, however, is not without a precedent, since the last chapter of Rasselas is "a conclusion in which nothing is concluded." In this *plan* the author introduces himself to our notice as a chemist in the following strain:

"I well recollect the first chemical experiment I ever witnessed, which was performed by my distinguished instructor, Dr. Ure, in the Andersonian Institution, Glasgow, and went to prove that water is composed of two different sorts of air, or, as chemists term them, gases. I was delighted—I may rather say enraptured—with the clearness of the unquestionable and irresistible proofs, and lay awake the greater part of the following night, mentally repeating and re-repeating the premises and the inferences therefrom, and wishing (vainly indeed, but I could not help it) that it had fallen to my lot to have made Cavendish's discovery of the extraordinary facts of the composition of water. My mind was roused into a train of thinking entirely new; and, as I pondered over the various circumstances, fresh combinations arose in numerous and delightful succession. I know not, indeed, that I ever experienced greater pleasure afterwards, even *when I had mastered the rudiments of the science*, and heard from another highly distinguished chemist, Dr. Thomson, whose pupil I also had the good fortune to be, the interesting doctrine of proportional combination, than I first felt from seeing the composition and decomposition of water."

We know the merits of Dr. Ure too well to believe that he ever attempted to prove anything so truly absurd as that water is composed of two gases. It is composed of oxygen and hydrogen, but not of oxygen and hydrogen *gases*. Almost any work on chemistry would have informed Professor Rennie that, when water is produced by the union of oxygen and hydrogen, the two gases disappear. If he does not know the difference between oxygen and oxygen gas, and the difference between any other gas and its base, he should not write for *beginners;* and that he does *not* know the difference, is further confirmed by his telling us that

"Chalk is composed of carbonic acid *gas* and lime."

"Peroxide of mercury is composed of oxygen *gas* and mercury, &c."

At page 3 the professor attempts to draw a distinction between chemistry and natural philosophy, or physics.

"In chemistry, we study changes that take place on a small or minute scale; and in physics, those on a great or extensive scale."

Accordingly, when we study the changes that take place in forming a few grains of any substance in a wineglass, we study chemistry; but if we study the same changes on a great or extensive scale, as in forming some tons of the same substance for the purposes of commerce, we then study physics. "A second Daniel come to judgment!" We find a very different distinction made by other writers between chemistry and natural philosophy; but the fact is, the professor has drawn no distinction at all, and therefore, instead of enlightening *beginners,* he is only leading them astray.

Page 20. "That the air has weight is known to every body who has felt the wind blow, or seen a ship sail."

Force is not weight. Were the wind to blow a professor out of his chair, it would no more prove that air has weight, than lightning rending the sturdy oak proves, what never has been proved, namely, that electricity is a ponderable body.

Page 26. "Nitrogen gas constitutes the basis of nitre, the well known substance popularly termed saltpetre."

Now, potass is the basis of the salt in question. Nitrogen is one of the simple bodies; and Professor Rennie is another, not to know that no simple body has the power of uniting with an acid, so as to form the basis of a salt.

Page 33. "The bleaching properties of sea air evidently depend upon minute portions of sea water, derived from the spray, and carried about by the winds, and as this contains a considerable portion of the principal substance, chlorine, used in artificial bleaching, the cause of the effect becomes evident."

Not quite so evident; for the chlorine, being in a state of strict chemical combination, is devoid of the property of bleaching, which it possesses in a state of separation. If the sea air has the property of changing the colour of black and other dyes, in consequence of being impregnated with saline particles, this is not a bleaching property; for bleaching is a total destruction of colour.

At page 35 we are made acquainted with the art of drying water.

"To obtain common salt, it is only necessary to dry (if I may thus use the term) a great quantity of sea water, which is done by exposing it to heat sufficient to drive off, in vapour, all the common or fresh water, in which the salt is originally dissolved."

In more passages than the above, the professor dwells a great deal upon the common water contained in sea water, and of *drying* sea water. Water is water, consisting always of oxygen and hydrogen, and all waters merely differ from each other in respect to the salts they hold in solution. If the water be driven off from such salts by evaporation, we should think the process that of drying the salts, and not the water. Drying water is about as practicable as extracting sunbeams from cucumbers; an art practised only at Laputa.

At page 62 we are informed that

"Carbonic acid has been proved to be the only substance in the diamond, which is pure carbon."

The professor "remembers a mass of things, but nothing distinctly." The diamond is the purest carbon met with in nature. The well known substance called charcoal is also carbon, but mixed with impurities. The diamond and charcoal, though differing so widely in appearance, are proved to be identically the same, by their both uniting with oxygen during combustion, and forming carbonic acid gas; for, as all lines drawn from the centre of a circle are equal to each other, so the same chemical compound must always consist of the same chemical constituents. The diamond, then, does not contain carbonic acid, but only forms it by combining with oxygen, precisely as charcoal does; and the reason that the two substances in question are so widely dissimilar in appearance is no doubt owing to their particles being in a different state of aggregation. The same may be said of chalk, limestone, marble, and the beautiful varieties of crystallized carbonate of lime; all of which, though differing in appearance, are identically the same in respect to their chemical constituents, which are in each case 22 parts of carbonic acid, and 28 parts of lime.

In describing the mode of procuring nitric acid from nitre by means of sulphuric acid, our author tells us (page 76) that

"The *sulphur* of the sulphuric acid leaves it, and lays hold of the *potass* in the nitre, while the *oxygen* and *nitrogen gases*, *thus set free*, unite and pass into the *adopter* in the form of red or orange fumes, which will condense and mix with the water in the bottle. The red or orange fumes which come over, and which become colourless as the process advances, but redden again towards the close, consist of the nitric acid, which is itself transparent, and what is termed *nitric gas*, (technically deutoxide of nitrogen; by Sir H. Davy, nitric oxide,) which is also transparent; but, when it meets with common air, or oxygen, it becomes coloured red, by forming nitrous acid with oxygen."

It is not necessary to take up our readers' time by giving the true and very simple theory of the separation of nitric acid from nitre, because it will be found in almost any respectable treatise on chemistry. Neither is it necessary to say any thing respecting the absurdity of *mixing* the nitric acid with water as it distils over, when the object is always to have it as free from that liquid as possible. This strange account is accompanied with a woodcut of the apparatus usually represented in chemical works, under the directions for making nitric acid on a small scale; and, both in the text above quoted, and the description of the said woodcut, our author clearly shews that he does not know the difference between an *adopter*, which is to fit two portions of apparatus together, and a *receiver*, which is to receive any liquid distilled.

Page 85. "Orpiment is a compound of sulphuric acid and arsenic."

Here is a discovery of a new salt, for we know not what else to call it; but, as orpiment happens to be composed of sulphur and arsenic, and as no acid is known to combine with arsenic, because as yet no true oxide of that metal is known; and, as acids only combine with the oxides of metals, and not with metals themselves, in all probability this new salt of the professor's, like several other of his compounds, has been formed at the writing-desk by means of scissors and paste.

"We are told in most books, that the *nature* of light or heat is unknown, meaning plainly by *nature* the fanciful nothing, otherwise termed substance or essence; for the properties of light and heat are as well known as those of water or iron. The same fanciful dreams have given rise to the impertinent discussions whether light, heat, &c. be material; that is, whether they have a frame-work of the long, broad, thick, impenetrable, and fanciful nothing, which nobody ever saw or felt, called *matter;* or whether they be mere motions, existing of course, without any thing being moved, and like no other motion. The impertinence and folly is carried further by those who argue for light, heat, &c. being supported on this fanciful frame-work, as they hesitate not to call them fluids, though they cannot be shown to possess one property in which they in the least resemble water or other fluids. It is surely high time that all such visionary stuff were banished from sciences pretending to be supported on rational proofs; yet I scarcely know a single scientific work that is not more or less contaminated therewith."

In the above passage our author boldly asserts what he almost immediately flatly contradicts: he states, that "*the properties of light and heat are as well known as those of water;*" and then, in the very next sentence, he tells us that "*they cannot be shown to possess one property in which they in the least resemble water, or other fluids.*" What Mr. Rennie may intend beginners to understand by this mode of reasoning, we shall not trouble ourselves to inquire; but all philosophers are agreed, that the properties of the imponderable bodies, light, heat, &c., are not precisely known, although we may in some measure be acquainted with the effects of such bodies on ponderable materials. That they are fluids there cannot be the least doubt; and it is remarkable, that the Hebrew word AUR, *light*, is derived from AR, *to flow.* Whatever has the power of flowing is designated by the term *fluid*, which every body knows is derived from the Latin verb *fluo,* to flow; and that light flows from the sun, caloric flows from a hot to a cold body, electricity flows from the clouds to the earth, &c., no one except Professor Rennie would attempt to deny. But the fact is, that he is not aware of the difference between a fluid and a liquid; for, at page 24, we find him speaking of "*liquid* quicksilver." Instead of finding fault with the terms employed by the best authors, he would do well first to make himself acquainted with their real meaning, of which, in many instances, he has not the faintest notion, although he has sometimes attempted to give their derivation. What can he possibly know of derivations, when he tells us that the Greek *α privative* signifies *against?*

At page 99, we are informed that

"If a small bit of the new metal potassium be placed on ice or snow, it will combine with the oxygen therein contained, *and at the same time force out its heat in form of a very brilliant flame*, proving that the ice or the snow contains a great deal of heat."

We shall not trouble our readers by repeating the well-understood theory of the decomposition of water or ice by potassium, because it is presumed they are well acquainted with it; but we beg to remind them, that our author is the first to speak of *heat burning with a brilliant flame.*

Mr. Rennie has a strong aversion to all terms used by scientific writers. For instance, *chemical attraction*, or *affinity*, he calls chemical *preference;* and, in his "Laws of Chemical Combinations," (which, by-the-bye, differ from those laid down by all other authors, ancient and modern), he gives the following tirade respecting *chemical preference:*

"Some chemical substances show a very marked preference of each other, and no less a marked antipathy to some other substances, as much as a brood of chickens will keep close to their mother, and will scuttle away for fear of a kite. The chemical preferences in question are observable between substances the same or similar, and between substances that are dissimilar."

We think the generality of tyros are sufficiently advanced to inform this learned purveyor of knowledge that there is no such thing as chemical affinity—or, if he likes, *preference*,—between substances the *same* or *similar;* for, that all chemical compounds are composed of *dissimilar* bodies, is one of the very first chemical axioms that a pupil becomes acquainted with. We will therefore leave our author, and his *preferences* between substances the same or similar, and pass on to *preferences between dissimilar subjects*, at page 129, where he says:

"I have already stated that an alkali will turn a vegetable blue colour to green, and an acid will turn it red; but when an alkali and an acid, having a mutual preference, combine and form a salt, this salt will not change the blue colour either to red or green, and will not taste either acid or alkaline."

Poor Professor Rennie! He is born to err, as the sparks fly upwards. Now if, instead of puzzling his brains to invent new terms, which will be read only to be forgotten, he had examined the carbonates of potash and soda, he would have found that they not only turn a vegetable blue to green, but also have a strong alkaline taste; and, had he pursued his inquiries still further, he would have discovered that nitrate of lead, and other salts, will turn a vegetable blue red, although the bases of such salts,* and their acids, have a mutual preference, as he calls it; but "there are more things in heaven and earth than are dreamt of in his philosophy."

At page 137, we find him stultifying the minds of beginners, by informing them that

"The *doctrine of proportional combinations* is technically *the atomic theory*."

But perhaps he may hereafter learn that the atomic theory is merely based upon the doctrine of proportional combinations, and that, should the theory in question be hereafter disproved, still the truth of the doctrine of proportional combinations cannot be injured thereby. We would advise the professor to read authors more attentively before he borrows from them: he ought at least to understand what he copies. But he appears to go on the plan of "here a little, and there a little," until his numerous shreds and patches are made to occupy the space of a book.

Our author has attempted to say something respecting "the language of chemistry;" but here again he only proves that he knows just as much of the meaning of the names as he does of the bodies to which they belong.

"Oxygen forms oxides, acids, and water."

"Hydrogen forms *hydrates*, *hydracids*, and *hydrogurets*." (P. 147.)

Hydrogen, like oxygen, can form nothing of itself in the shape of a chemical compound; but, with a few bases, it forms *hydrurets* or *hydrogurets*, these terms being synonymous. It never forms compounds called *hydrates;* for these are the results of these combinations in which water becomes an ingredient in its combining proportion, or a multiple of that proportion; or, in other words, becomes chemically combined.

"Nitrogen along with oxygen forms *nitrates* and *nitrites*." (P. 147.)

* The oxides of the ordinary metals, in combining with acids to form salts, play the part of the alkalies alluded to above; and, were such oxides soluble, they would no doubt have an alkaline reaction on vegetable blues and yellows.

Nitrogen, along with oxygen, forms five compounds, not one of which is either a *nitrate* or a *nitrite;* as Mr. Rennie might have learned, had he paid proper attention to his instructors, Drs. Ure and Thomson, instead of leaving Glasgow prematurely, under the whimsical supposition that he "*had mastered the rudiments of the science.*"

"Chlorine forms *chlorides* and *chlorurets*, and along with oxygen forms *chlorates.*" (P. 147.)

Chlorides are chlorurets, and chlorurets are chlorides, the two terms being strictly synonymous. Oxygen and chlorine combine to form four compounds, not one of which is called a *chlorate.* It were tedious to proceed further in the investigation of our author's nomenclature, or we might adduce a very great number of other instances in which errors similar to the above are advanced to smooth the path of chemistry to *beginners.* That Mr. Rennie never saw any of the compounds he speaks of is but too evident; for he tells us that *Protochloride of mercury*" (or calomel, which every old woman knows to be a white substance,) is "popularly *cinnabar* or *vermilion;*" and that "*perchloride of mercury*" (corrosive sublimate) is "popularly calomel." (P. 151.)

We might have noticed a curious error of our author, who asserts, at page 105, that the blood is "carried to the heart, by whose motion or beating it is thrown into a large vein, and carried to the lungs;" but we are tired of the painful task of correcting perpetual blunders. Indeed, we fear that some of our readers will blame us for wasting so much space on so short and trifling a work, while others will charge us with uncalled-for severity towards an industrious and well-meaning man; but we must answer both classes of objectors, by observing, that the errors which would be inconsequential if proceeding from plain Mr. Rennie, are dangerous when sanctioned by the authority of a professor of King's College; and, moreover, we thought it the duty of honest reviewers to interpose some check to the unbounded itch for writing, which is advantageous to no one but the printer, which makes talent ridiculous, and industry useless.

